# The short and long-term efficacy of nurse-led interventions for improving blood pressure control in people with hypertension in primary care settings: a systematic review and meta-analysis

**DOI:** 10.1186/s12875-024-02380-x

**Published:** 2024-04-27

**Authors:** Masami Ito, Aran Tajika, Rie Toyomoto, Hissei Imai, Masatsugu Sakata, Yukiko Honda, Sanae Kishimoto, Memori Fukuda, Noboru Horinouchi, Ethan Sahker, Toshi A. Furukawa

**Affiliations:** 1 Departments of Health Promotion and Human Behavior, Kyoto University Graduate School of Medicine / School of Public Health, Yoshida Konoe-Cho, Sakyo-Ku, Kyoto, 606-8501 Japan; 2 Department of Community Medicine, Nagasaki University Graduate School of Biomedical Sciences, Nagasaki, Japan; 3 Department of Social Medicine, National Centre for Child Health and Development, Tokyo, Japan; 4 Department of Health Informatics, Kyoto University Graduate School of Medicine / School of Public Health, Kyoto, Japan; 5 Department of General Medicine, Oita University Faculty of Medicine, Oita, Japan; 6 Population Health and Policy Research Unit, Medical Education Center, Graduate School of Medicine, Kyoto University, Kyoto, Japan

**Keywords:** Hypertension, Nurse, Nurse-led intervention, Nursing care, Nursing intervention, Patient care management, Health education, Primary care, Systematic review

## Abstract

**Background:**

Previous systematic reviews suggest that nurse-led interventions improve short-term blood pressure (BP) control for people with hypertension. However, the long-term effects, adverse events, and appropriate target BP level are unclear. This study aimed to evaluate the long-term efficacy and safety of nurse-led interventions.

**Methods:**

We conducted a systematic review and meta-analysis. We searched the Cochrane Central Register of Controlled Trials, PubMed, and CINAHL, as well as three Japanese article databases, as relevant randomized controlled trials from the oldest possible to March 2021. This search was conducted on 17 April 2021. We did an update search on 17 October 2023. We included studies on adults aged 18 years or older with hypertension. The treatments of interest were community-based nurse-led BP control interventions in addition to primary physician-provided care as usual. The comparator was usual care only. Primary outcomes were long-term achievement of BP control goals and serious adverse events (range: 27 weeks to 3 years). Secondary outcomes were short-term achievement of BP control goals and serious adverse events (range: 4 to 26 weeks), change of systolic and diastolic BP from baseline, medication adherence, incidence of hypertensive complications, and total mortality.

**Results:**

We included 35 studies. Nurse-led interventions improved long-term BP control (RR 1.10, 95%CI 1.03 to 1.18). However, no significant differences were found in the short-term effects of nurse-led intervention compared to usual care about BP targets. Little information on serious adverse events was available. There was no difference in mortality at both terms between the two groups. Establishing the appropriate target BP from the extant trials was impossible.

**Conclusions:**

Nurse-led interventions may be more effective than usual care for achieving BP control at long-term follow-up. It is important to continue lifestyle modification for people with hypertension. We must pay attention to adverse events, and more studies examining appropriate BP targets are needed. Nurse-led care represents an important complement to primary physician-led usual care.

**Supplementary Information:**

The online version contains supplementary material available at 10.1186/s12875-024-02380-x.

## Introduction

Hypertension is an important issue in public health, leading to serious health complications [1]. It is a major cause of premature death worldwide and one of the greatest risk factors for the global burden of disease, afflicting 1.28 billion people worldwide [2]. The number of people with hypertension is increasing as the world population grows and ages.

The American College of Cardiology/American Heart Association, European Society of Cardiology/European Society of Hypertension, and Japanese Society of Hypertension updated their guidelines recommending that BP targets for hypertensive patients should be lower than the goals formerly set by the conventional guidelines, namely as BP targets < 130/80 mmHg [3–5]. However, it can be difficult to keep patient BP targets lower, and the benefits of such strict BP regulations are limited to reduced health complications. The evidence is not clear on whether lower BP targets reduce total mortality or eschew serious adverse events [6].

Previous reviews have found that allied health professional-led interventions can improve BP control for patients with hypertension [7, 8]. Patients with hypertension need to adhere to medications, and importantly, they must modify their lifestyle (e.g., sodium restriction, smoking cessation, and alcohol use reduction) [5, 9]. However, patients with hypertension have been found to be less likely to practice lifestyle modifications [10]. In this context, nurse-led hypertension interventions may be particularly helpful by including more individualized care, enhancing care quality, and improving patient self-control due to increased patient contacts, and thus contribute to better BP control [11]. Existing systematic reviews have not examined adverse events and their results are hard to interpret because their control groups include both usual care by physicians and no intervention, and they mix outcomes at both the long and short term [11]. Importantly, the appropriate time it takes for patients to present with clinically meaningful BP control has not been identified in the evidence base. Because behavior change varies by patient, nurses and physicians would benefit from identifying reasonable expectations for patient BP control goals.

Therefore, the present study aimed to evaluate both the short- and long-term efficacy and safety of nurse-led interventions to improve BP control for people with hypertension. Secondly, the present study explores whether adverse events reported in nurse-led interventions could be due to strict BP control targets.

## Methods

The present study is a systematic review and meta-analysis. We conducted a review according to the Cochrane Handbook for Systematic Reviews of Interventions ver. 6.1 [12]. We followed the PRISMA 2020 guidelines [13, 14]. This study has been registered with PROSPERO [CRD42021246085].

### Study selection

We included nurse-led BP control interventions in addition to primary care physician-provided usual care. Types of interventions were aimed at improving BP control as lifestyle modifications. The model of primary care settings was defined as the first point of contact in the healthcare system [15], “home-based and community-based care, primary care in long-term care facilities, step-down units for rehabilitation in local hospitals, dedicated emergency care units at comprehensive health centers and first level hospitals” [16]. Nurses included any professionals with relevant state qualifications. There were no restrictions in nurses’ place of work, work settings, age, or experience. We included usual care only in primary care by physicians as the comparators. Therefore, we evaluated the added effects of nurse-led interventions to usual care.

We included all published and unpublished randomized controlled trials (RCTs). For cross-over studies, only the first phase was included. We also included cluster randomized controlled trials if the intra-cluster (or intraclass) correlation coefficient (ICC) could be estimated [17]. Participants included adults aged 18 years or over with hypertension according to each author’s use of any standardized diagnostic criteria. There were no restrictions on the presence or absence of hypertensive complications. No restrictions were made in terms of sex/gender, ethnicity, or country. We excluded studies of pregnant women.

### Primary and secondary outcomes

We defined short-term as less than six months (range: 4 to 26 weeks) and long-term as more than 6 months (range: 27 weeks to 3 years) separately. We also used the point closest to one year when there were multiple time points, such as one or three years. The primary outcomes were (1) long-term achievement of BP control goals and (2) long-term serious adverse events. BP control goal was to keep BP targets or lower. Clinicians would consider long-term BP achievement important. We used the number of serious adverse events as defined and reported by the trial authors. The secondary outcomes were (1) short-term achievement of BP control goals, (2) short-term serious adverse events, and all the following both at long and short term: (3) change from baseline of systolic blood pressure (SBP)/diastolic blood pressure (DBP), (4) rate of antihypertensive drugs prescribed, (5) medication adherence, (6) incidence of hypertensive complications including cardiovascular events and strokes, and (7) total mortality.

### Search strategies

We searched the Cochrane Central Register of Controlled Trials (CENTRAL) (1946 to 31 March 2021), PubMed (1966 to 31 March 2021), and CINAHL (1982 to 31 March 2021), as well as the following three Japanese article databases: Ichushi-Web (1946 to 31 March 2021), Current Index to Japanese Nursing Literature (1987 to 31 March 2021), and CiNii Articles (1923 to 31 March 2021). All searches were conducted on 17 April 2021. We did an update search in PubMed (2021 to 30 September 2023) and CENTRAL (1 April 2021 to 30 September 2023) on 17 October 2023. We did not use any language restrictions to minimise the language bias [18]. Search strategies for each database are listed in the supplementary materials (See Supplemental Table S1). We also checked the references of previous studies on this topic. We asked original authors to provide the details of ongoing studies, conference abstracts, oral sessions, and unpublished data if needed.

Pairs of review authors (M.I., H.I., M.S., Y.H., R.T., S.K., and N.H.) independently identified the titles and abstracts from potentially relevant studies to be retrieved in the first stage of screening using Rayyan software [19]. We removed duplicates and obviously irrelevant reports. Next, we retrieved the full article for potentially relevant reports. The pair of review authors independently decided which studies met all eligibility criteria. The reviewers discussed any disagreement about inclusions. We consulted a third review author (A.T.) of our team if we could not resolve disagreements. We also used DeepL Translator [20] in the second stage of screening when we needed to translate other languages into English. We have finally asked the native language users to check the articles.

We constructed a data extraction form using Excel spreadsheets for this review. The following characteristics were extracted from included studies: study name, year of publication, study designs, type of setting, country, diagnostic criteria used, the group-average demographics, details of the intervention, outcome measures, BP goals, and sphygmomanometer type (See Tables 1 and 2 for details). Pairs of the review authors (M.I., H.I., M.S., Y.H., R.T., S.K., and N.H.) independently extracted data from all included studies. They also discussed any disagreements and noted their decisions. We involved a third review author (A.T.) in cases of disagreement.

**Table 1:** Summary of study characteristics

Study characteristic (*N* = 35)	Number of articles, (%)	
Type of study		
Individual RCTs	31	(88.6)
Cluster RCTs	4	(11.4)
Region		
North America	16	(45.6)
South America	1	(2.9)
Europe	7	(20.0)
Asia	10	(28.6)
Oceania	1	(2.9)
Care Setting		
Primary care clinic	8	(22.8)
General practices	1	(2.9)
Community health care centers	11	(31.4)
Home-based and community-based care setting	5	(14.3)
Hospital (Department of general internal medicine)	3	(8.6)
Military medical center	1	(2.9)
Company	2	(5.7)
Mixed settings	4	(11.4)
Baseline characteristic		
Hypertensive complications		
Yes	19	(54.2)
No	1	(2.9)
Unclear	15	(42.9)

**Table 2:** Study characteristics in the included studies

Study			Population						Intervention	Outcomes	
Author, year of publication	Length of RCT,weeks	County, settings	Sample size No. cluster/No. participants		Age, mean	Female, %	Hypertensive complications	Baseline mean BP SBP/DBP, mmHg	The details of care^a^	Achievement of BP control goals at the primary outcome (SBP/DBP mmHg)	Sphygmomanometer type
Amado GE, 2011 [21]	52	Spain,	18	481	63.4	64.2		139.3/82.2	Usual care		
		Community health care centers	18	515	63.3	67.7	Unclear	140.9/82.5	Personalized information by a trained nurse, and written leaflets. The disease, healthy lifestyle habits, and messages targeted to each group of antihypertensive drugs used (mechanism of action, dosage, what to do if a pill is missed, adverse effects, and other recommendations) were contained	-	Mercury
Beune EJAJ, 2014 [22]	26	Netherlands,	2	71	54.6	44.0		155.2/89.6	Usual care		
		Community health care centers	2	75	53.3	61.0	Unclear	156.7/91.0	Nurse-led culturally appropriate HT education sessions: counselling and educational materials; if applicable, referrals to neighborhood facilities, such as walking clubs and health food stores, that support patients in adopting healthier lifestyles	-	Automated
Bogner HR, 2013 [23]	12	USA,	-	30	65.8	60.0		139.4/76.4	Usual care		
		Community-based primary care practices	-	30	68.3	70.0	+	133.6/76.5	An integrated care by licensed practical nurses: provision of an individualized program to improve adherence to antidepressants and antihypertensives, and integration of depression treatment with HT management	-	Automated
Boswort HB, 2009 [24]	104	USA,	-	159	62.0	64.0		124/70	Usual care		
		Primary care clinics	-	160	60.0	67.0	+	124/71	Nurse administered telephone-based tailored behavioral intervention: included perceived risk of HT, memory, literacy, social support, patients’ relationships with their health care providers, and side effects of anti-hypertension medication; and focused on improving adherence to the following HT recommendations: the Dietary Approaches to Stop Hypertension (DASH) dietary pattern, weight loss, reduced sodium intake, regular moderate intensity physical activity, smoking cessation, and moderation of alcohol intake	< 140/90 mmHg, diabetes: < 130/80 mmHg	Automated
			-	159	61.0	62.0		126/72	A combination of medication and behavioral management: received a home BP monitor, training on its use, and the bi-monthly nurse-administered behavioral self-management intervention		
			-	[158]	[62.0]	[71.0]		[126/72]	[Home BP monitoring]		
Boswort HB, 2011 [25]	78	USA,	-	147	64.0	4.0		128/78	Usual care		
		Home-based and community-based care settings	-	148	63.0	8.0	+	129/77	Nurse-administered, behavioral management intervention: used an intervention software application. cf. BosworthHB2009	< 140/90 mmHg,	Automated
			-	149	64.0	7.0		132/78	Nurse-administered, physician-directed medication management intervention using a validated clinical decision support system	diabetes: < 130/80 mmHg	
			-	147	63.0	14.0		127/77	Combined behavioral management and medication management intervention		
Cakmak V, 2021 [26]	13	Turkey,	-	50	55.1	64.0		130.0/82.4	Usual care		
		Community health care centers	-	50	55.4	68.0	-	127.7/83.3	One-on-one training in the form of one-on-one verbal expression and question–answers. Colorful education booklets were given to the patients for reading at home	-	Unclear
Dejesus RS, 2009 [27]	26	USA,	-	18				148.4/73.5	Usual care		
		Primary care clinics	-	17	< = 60y 13people,	52.0	+	152.41/73.4	A class focusing on HT in diabetes and BP checked by a registered nurse	-	Automated
			-	19	> 60y 41people			148.3/72.0	A class focusing on HT in diabetes, BP checked by a registered nurse and received additional instruction on home BP monitoring from registered nurse		
Duong D, 2004 [28]	104	USA,	-	57	61.9	36.8		136.4/78.7	Usual care		
		Military medical centers	-	90	57.0	45.6	Unclear	133.0/74.8	A comprehensive educational-behavioral intervention program: knowledge regarding HT, adherence to recommended therapies, communication skills with health care providers, lifestyle behaviors, blood pressure control, and satisfaction with care by clinical nurse specialist; Based on the PRECEDE-PROCEED model, the goals of lower high blood pressure and improve patient satisfaction with available health care	< 140/90 mmHg	Mercury
Feldman PH, 2020 [29]	52	USA,	-	165	66.5	58.8		154.3/86.2	Usual home care (UHC)		
		Home-based and community-based care settings	-	165	66.9	59.4	+	154.9/85.7	UHC, and a 30-day nurse practitioner (NP) transitional care program The NP was responsible for (1) conducting a comprehensive health assessment; (2) communicating with the patient's physicians and UHC nurse; (3) monitoring BP; (4) ensuring appropriate medication and behavioral regimens; (5) collaborating with patients and caregivers to overcome barriers and adhere to a tailored, culturally sensitive self-management plan; and (6) addressing patients' social support needs through referral to appropriate community resources	-	Automated
			-	165	66.4	52.7		154.2/85.5	UHC, and the intensive 30-day NP intervention plus a 60-day of coaching/self-management support from a home health aide specially trained to be a heath coach		
Feldman PH, 2016 [30]	52	USA,	-	292	63.3	66.0		154.5/87.6	Usual home care		
		Home-based and community-based care settings	-	267	65.1	65.0	+	155.7/86.5	Usual home care, and field nurses received the Seventh Report of the Joint National Committee on Prevention, Detection, Evaluation and Treatment of High Blood Pressure (JNC7) guide, BP monitor with instructions	< 140/90 mmHg	Automated
			-	286	64.4	70.0		154.1/87.0	Augmented Usual home care: incorporated all components of the basic intervention and of usual home care. For stage 2 HT, usual home care plus provided HT medication assessment, monitoring, education and self-management support by nurse and a health educator	< 130/80 mmHg for patients with diabetes or kidney disease: JNC7 guidelines	
Given CW, 1984 [31]	39	USA,	-	24	45.9			142.8/93.8	Usual care		
		Mixed settings (family-practice and office practice)	-	62	47.7	Unclear	Unclear	145.0/93.5	The problem-solving intervention upon patients’ beliefs about and control of BP by nurses. Educational handbook was used describing the risks of HT	-	Unclear
Kabayama M, 2021 [32]	26	Japan,	-	25	64.6	0		141.6/82.1	Usual care		
		Primary care clinics	-	28	63.4	0	+	140.4/84.2	Health guidance provided face-to face, aiming at optimal alcohol habits by trained nurses	-	Automated
Kastarinen MJ, 2002 [33]	104	Finland,	-	355	54.2	54.0		148/91	Usual care		
		Community health care centers	-	360	54.4	52.0	+	149/91	Systematic health counselling given by local public health nurses. This intervention goals for the study subjects were: (1) normal weight (BMI, 25 kg/m2); (2) daily sodium chloride intake less than 5 g; (3) alcohol consumption fewer than two drinks per day; (4) to exercise at moderate intensity at least three times per week for 30 min; and (5) to stop smoking if a smoker	-	Mercury
Kes D, 2021 [34]		Turkey,	-	46	52.2	55.3		155.3/94.2	Usual care		
	12	Community health care centers	-	46	54.9	51.3	Unclear	156.3/95.1	Nurse-led support by regular text messages and phone calls to take prescribed medication	< 140/90 mmHg	Automated
Kolcu M, 2020 [35]	24	Turkey,	-	38	75.6	43.2		119.2/75.1	Usual care		
		Nursing homes	-	38	75.6	48.6	Unclear	129.2/79.7	Nurse-led HT management program included the Dietary Approach to Stop Hypertension (DASH) diet, and dietary approach; The training program (Health education and motivational meetings) consisted of individual and group interventions together with actions taken at the institutional level	-	Mercury
Leiva A, 2014 [36]	52	Spain,	-	115	66.7	46.1		155.5/83.6	Usual care		
		Community health care centers (primary care centers)	-	115	64.5	41.0	+	156.3/84.7	A multifactorial adherence-based intervention including nurse-led motivational interviews based on the Heath Belief Model, pillbox reminders, family support, BP self-recording, and simplification of the dosing regimen by a pharmacist	-	Automated
Litaker D, 2003 [37]	104	USA,	-	78	60.6	58.0			Usual care		
		Hospital (Department of General Internal Medicine)	-	79	60.5	59.0	+	Unclear	The intervention focused on chronic disease management by physician-nurse practitioner teams, and the use of clinical practice algorithms, patient education on disease self-management strategies, and regular monitoring and feedback delivered primarily by the nurse practitioner	< 130/85 mmHg	Unclear
Logan AG, 1983 [38]	52	Canada,	-	97	49.3	30.9		154.9/103.3	Usual care		
		Companies	-	97	50.8	23.7	Unclear	154.3/103.7	The treatment by their family doctor and the occupational health nurse (OHN) at their place of employment; The process of care of HT patients by the OHN (i.e., referral to a physician, initiation of appropriate therapy, and long-term maintenance of treatment). The OHN was instructed to ensure that employees saw their family physician and to assist employees without a physician to obtain one	Baseline DBP > = 95 mmHg: < DBP 90 mmHg, DBP 91-94 mmHg: -6 mmHg	Mercury
Ma C, 2014 [39]	24	China,	-	60	58.4	53.3		150.3/88.7	Usual care		
		Community health care centers	-	60	59.2	48.3	Unclear	153.2/89.0	Counselling based on motivational interviewing and social cognitive theory, and designed to address HT care. The nurses asked the patients to record a daily diary, and the content included information on adherence to medication, dietary habits, physical activity, drinking and smoking, illness perception, physical health, and mental health	-	Automated
Ma Y, 2022 [40]	12	China,	-	105	61.5	54.3		150.6/93.3	Usual care		
		Community health care centers	-	105	59.6	51.4	+	149.7/93.8	Six individual weekly education and consultation sessions provided by a nurse in the first 6 weeks and a smartphone application for 12 weeks. The sessions consisted of health education, individual self-care planning, daily records of physical health status and lifestyle behavior, and an automated weekly health report	< 140/90 mmHg	Automated
Matteida Silva ÂT, 2020 [41]	52	Brazil,	-	47	49.2	72.3		134.7/85.1	Usual care		
		Primary care clinics	-	47	49.4	83.0	+	135.2/85.6	Nursing case management model included:(1) nursing consultations; (2) telephone contact to re‐evaluate the patients’ healthcare plans, remind them of their consultation agendas, provide guidance for the adoption of healthy habits and disease control with WhatsApp®; (3) home visits; (4) health education; and (5) appropriate referrals Group activities were focused on topics such as the development of healthy habits, physical activity, treatment adherence, blood pressure measurement, and chronic complications	< 140/90 mmHg	Automated
Miao J-H, 2020 [42]	16	China,	-	78	66.8	53.9			Usual care		
		Community health care centers	-	78	68.9	49.0	+	Unclear	The nurse-led HT management involved home visits guided by the Omaha System, telephone follow-ups and referrals; included smoking cessation, alcohol restriction, salt restriction, regular physical activity, and home BP monitoring. The trained nurses performed relevant interventions that included teaching/guidance/ counseling in lifestyle modification changes, treatment and procedures such as timing and dosage adjustment as well as drug interactions and physical activity, and case management	-	Unclear
Okuda N, 2010 [43]	22	Japan,	-	18	46.4	0		136.8/88.4	Usual care		
		Companies	-	18	45.1	0	Unclear	135.2/90.0	Lifestyle counseling program by the occupational health nurse included salt restriction, potassium-intake addiction, physical activity, alcohol restriction, and weight loss	-	Unclear
Persell SD, 2018 [44]	52	USA,	4	305	53.0	72.0		141.5/86.9	Usual care		
		Community health care centers	4	313	51.6	67.3	+	145.5/89.5	Nurse-led medication management education and support. Nurses assessed patients’ knowledge of their chronic conditions, addressed misconceptions, and reinforced the role medications play in disease control; used the electronic health records (EHR) tools to identify potential medication errors (e.g., duplicates, internal discrepancies) and identify areas for monitoring and follow-up	< 140/90 mmHg	Automated
			[4]	[302]	[53.6]	[66.8]		[148.6/89.1]	[EHR-based medication management tools alone]		
Rudd P, 2004 [45]	20	USA,	-	76	56.0	9.0			Usual care		
		Primary care clinics	-	74	50.0	10.0	+	Unclear	Usual care supplemented by nurse management for HT. The nurse care manager conducted baseline counseling on intervention patients’ correct use of the automated BP device, regular return of the automatically printed BP reports, tips for enhancing drug adherence, and recognition of potential drug side effects	-	Mercury
Ruppa TM, 2010 [46]	20	USA,	-	5	Unclear	60.0		151.2/82.4	Usual care		
		Mixed settings (home and senior living facilities)	-	10	Unclear	80.0	Unclear	136.0/74.4	The nurse-delivered adherence intervention consisted of 5 components: medication adherence and BP feedback, habit counseling, medication-taking skills and disease education by gerontological advanced practice nurse	-	Aneroid
Schroeder K, 2005 [47]	26	UK,	-	117	68.2	46.0		152.1/83.1	Usual care		
		Primary care clinics	-	128	67.9	44.0	+	149.0/83.7	Nurse-led adherence support (adherence-related training) to provide an opportunity for patients to talk about any problems with their blood pressure lowering medication	-	Unclear
Sen M, 2013 [48]	52	Sweden,	-	50	62.0	46.0		165.0/92.6	Usual care		
		Community health care centers	-	59	65.0	51.0	+	169.0/93.1	BP card summarized the essential targets of HT treatment, with an added semi-structured nurse counseling The nurse-led intervention was aimed at creating an individual plan with the intention of reaching target BP as well as focusing on evidence-based guidelines on lifestyle changes and supporting the modifications the patient wanted to work with	< 140/90 mmHg, Diabetes and kidney disease: < 130/80 mmHg, Patients who did not tolerate lower levels: < 150/90 mmHg	Automated
			-	[57]	[66.0]	[56.0]		[165.0/88.9]	[BP card only was introduced to the patient.]		
Swain MA, 1981 [49]	78	USA,	-	38				-/101.6	Usual care (Routine clinic care)		
		Mixed settings (a clinic located in a medical center hospital and the other in a Veterans Administration hospital.)	-	40	Unclear	Unclear	Unclear	-/100.1	Patient education: the instructional booklets (What you and your family need to know, Medication, Stress, Diet, and Activity)	-	Unclear
			-	37				-/97.1	Contingency contracting: received the instructional booklets and tests of knowledge, and wrote contracts with a nurse for targeted health goals and attendant rewards		
Ulm K, 2010 [50]	52	Germany,	-	98	65.1	52.0		156.3/92.7	Usual care		
		Primary care clinics (physician’s office)	-	102	65.8	41.2	+	155.9/90.8	The nurse-managed medical care programme with standardized BP measurement, self-measurement training, risk factor checks and advice on BP reduction by nurses trained intensively. Patients received a booklet on HT to receive individualized advice on how to change lifestyle factors and comply with the prescribed medication	-	Unclear
Wakefield BJ, 2011 [51]	52	USA,	-	107	67.9	4.0		133.8/-	Usual care		
		Home-based and community-based care settings	-	102	68.4	1.0	+	135.8/-	Low-Nurse managed home telehealth intervention directed by their physician. Patients were instructed to measure BP and blood glucose (BG) daily, and were asked “Have you taken all your medications as prescribed?”	-	Automated
			-	93	67.8	1.0		137.9/-	High-Nurse managed home telehealth intervention by the study team (nurses, a physician, and a certified diabetes educator) A branching disease management algorithm by the study team was programmed into the device and focused on diet, exercise, smoking cessation, foot care, advice for sick days, medications, weight management, preventive care, and behavior modification and lifestyle adjustments. Patients were instructed to measure BP and BG daily, and received both standard prompts each day and a rotation of questions and educational content		
Woollard J, 1995 [52]	18	Australia,	-	48	59.0	50.0		142/80	Usual care		
		General practices	-	52	58.0	44.2	Unclear	145/80	A lifestyle modification programme implemented by nurse: a low label of counselling; a single face-to-face appointment and then five telephone counselling sessions. The programme objectives were the same as the high level of counselling	-	Automated
			-	46	58.0	45.7		139/78	A lifestyle modification programme implemented by nurse: a high label of counselling; six face-to-face counselling sessions. The programme objectives were the same as the ow level of counselling The programme objectives encompassed the following: (1) Weight reduction following the Australian Nutrition Foundation guidelines (National Health and Medical Research Council (1991)); (2) In drinkers a reduction in alcohol intake to 1 standard drink a day (10 g) for women and 2 standard drinks daily (20 g) for men; (3) Salt restriction to less than 90 mmol/day; (4) Less than 30% daily energy dietary fat with restriction of saturated fat intake to 10%; (5) An increase in regular leisure time physical activity; and (6) Smoking cessation		
Zabler B, 2018 [53]	26	USA,	-	30	53.6	70.0		130.5/78.2	Usual care		
		Primary care clinics	-	29	53.9	55.2	Unclear	131.6/80.0	Ecological nurse case management intervention on perceived stress, self-efficacy, and self-management behaviors. The intervention is individualized to each patient and a key of ‘Mutual Self-Management Goal Setting’. The primary nurse actions included: self-management support, inclusive of both health education and behavior modification; technical procedures; care coordination; and/or surveillance	-	Unclear
Zang XY, 2010 [54]	13	China,	-	36	Unclear	Unclear		158.6/88.3	Usual care		
		Hospital (the medical clinic of the General Hospital)	-	36	Unclear	Unclear	Unclear	152.5/87.5	Behaviour interventions included the following; instructing patients to obey the types and doses of medicine and adjust the time of taking medicine according to chrono-therapeutics (the purposeful alteration of drug levels to match rhythms to optimise therapeutic outcomes and minimise size effects); and advising them to change bad lifestyles such as reducing physical activities when BP was rising, giving the plan for an anti-hypertensive diet and quitting cigarettes and alcohol, etc The psychological interventions (helping participants accommodate their mode to avoid the fluctuation of BP) under ambulatory BP monitoring	-	Automated
Zhu X, 2018 [55]	16	China,	-	67	69.0	52.2		149.7/83.5	Usual care		
		Community health care centers	-	67	69.0	49.3	+	153.9/82.6	Nurse-led HT management model including four components (delivery system design, decision support, clinical information system and self-management support) was developed. The trained nurses performed relevant interventions that included teaching/guidance/counseling, treatment and procedures, and case management. The self-management, such as salt intake control, regular engagement in physical activities, home BP monitoring management, and medicine storage, were included	-	Unclear

*BP* blood pressure, *DBP* diastolic blood pressure, *SBP* systolic blood pressure, *HT* hypertension

+ : Applicable, - : No report

^a^[ ]: excluded from the meta-analysis not to be nurse-led intervention

### Data synthesis and analysis

We used risk ratios (RR) and 95% confidence intervals (CI) for dichotomous outcome measures and mean difference (MD) and 95% CI for continuous outcome measures. We analyzed data with a generic inverse-variance approach for meta-analyses using Review Manager 5.4 software [56]. We used a random-effects model for all analyses because we anticipated clinical heterogeneity among various interventions by nurses. We used the results as reported by the authors abiding by the intention-to-treat (ITT) principle. We contacted the study authors to request missing data. We assumed that patient conditions remained unchanged when the relevant information was missing.

We analyzed outcomes from cluster randomized trials by reducing the size of each trial to its effective sample size with ICC. We contacted the original authors when ICCs were not reported. We estimated the effective sample sizes for cluster-randomized trials according to the Cochrane Handbook for Systematic Reviews of Interventions ver. 6.1 [12]. We borrowed ICCs (0.001) from other studies when the ICCs were not provided by the original authors [44]. We excluded studies from the meta-analysis when there were no events in both arms because such studies do not provide any indication of the direction or magnitude of the relative treatment effects [12]. In addition, we borrowed standard deviations (SDs) from similar studies when the SD for the change scores or the endpoint scores were missing [57, 58].

### Subgroup analysis

We performed the following pre-specified subgroup analyses: the target BP goals (< 140/90 mmHg, or 140/90 mmHg), regions (North America, South America, Europe, Asia, Africa, and Oceania), and settings (nurse interventions taking place in primary care clinics, district hospitals, in the communities, community health care centers, companies, nursing homes, and facilities for the elderly people, and others).

### Sensitivity analysis

We performed the following sensitivity analyses for the primary outcome to assess the robustness of our results: 1) we excluded studies at high risk of bias, 2) we regarded the dropouts as achieving BP goals, and 3) we excluded studies requiring borrowed ICCs.

### Assessment of heterogeneity

We interpreted heterogeneity using the *I*^2^ statistic as follows: 0-40% might not be important, 30-60% may represent moderate heterogeneity, 50-90% may represent substantial heterogeneity, and 75-100% represent considerable heterogeneity [12]. The *τ*^2^ indicated the spread of true intervention effects and was interpreted in comparison with its empirical distribution [59, 60].

### Reporting bias assessment

The pairs of review authors (M.I., H.I., M.S., Y.H., R.T., and S.K.) independently assessed the risk of bias of the included studies using the Risk of Bias Tool Ver. 2.0 (RoB 2.0), updated August 22, 2019 [61]. We assessed each risk of bias item at each intervention in the included studies with the following five domains: randomization process, deviations from intended interventions, missing outcomes, measurement of outcome, and selection of reported results. We graded each risk of bias as high, low, or some concerns for every domain. We also used the RoB 2.0 tool for cluster-randomized trials (updated March 18, 2021) if the included studies were cluster RCTs [61]. We assessed the risk of bias with a third review author (A.T.) when we disagreed. We assessed reporting biases by visual inspection of funnel plots and conducted Egger’s test for continuous outcomes with R Version 4.0.5 [62] when we had ten or more studies [63].

## Results

### Study selection

**Fig. 1:**
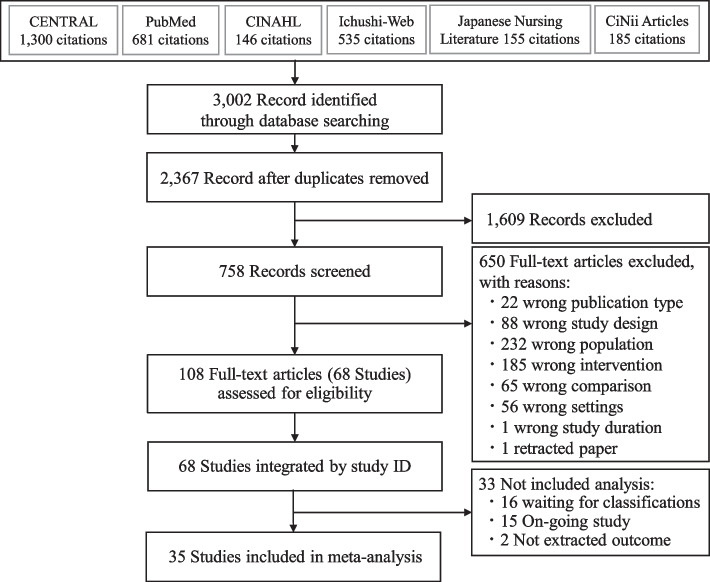
PRISMA flow chart

The search yielded 3,002 articles up to September 2023. We screened both titles and abstracts of 2,367 records after removing duplicates. In the first screening, 1,609 articles were excluded because of inapplicable titles and abstracts. We retrieved 758 full-text papers and included 108 articles. Finally, 35 studies were identified for the final quantitative synthesis in the meta-analysis. We discovered three new articles by update search in 2023. Figure 1 shows the PRISMA flow chart of the search and selection process results.

Table 1 summarizes the characteristics of the included studies. Half were conducted in North America. Four cluster RCTs were included out of 35 RCTs. Care settings varied, and half of the included studies were uncertain about hypertensive complications. Table 2 summarizes the characteristics of each of the included studies. Nurse-led interventions in the included studies covered many topics, such as counseling, personal and/or group education, behavioral management, coaching, remote monitoring, and motivational interviews. We tabulated which outcomes were available in each study (Supplement Table S2).

### Primary outcomes

#### Achievement of BP control goals at long-term

**Fig. 2:**
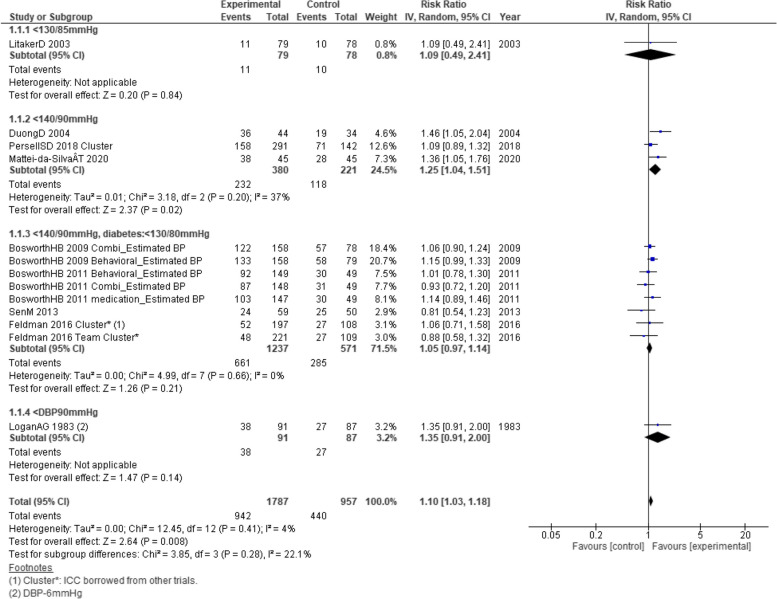
Forest plot of the achievement of BP control goals at long term (including the numeric data of the achievement of BP goals compared with usual care)

Results are presented in subgroups for each BP control goal (Fig. 2). Nurse-led intervention demonstrated greater achievement of BP control goals compared to usual care alone (RR = 1.10; 95% CI = 1.03, 1.18; *p* = 0.008; 9 included studies; 2,744 participants). The *I*^2^ was 4%, and *τ*^2^ was zero in the primary outcome, indicating low heterogeneity between the studies.

Included studies set various target BPs. One study [37] set below 130/85 mmHg, seven studies [24, 25, 28, 30, 44, 64, 65] below 140/90 mmHg, and one study [38] below DBP 90 mmHg with an unspecified SBP. However, the subgroup heterogeneity (*P* = 0.28, *I*^2^ = 22.1%) suggested no discernible subgroup differences. The subgroup analyses by region or care settings did not suggest important subgroup differences (Figures S3.1 and S3.2).

#### Serious adverse events at long-term

There was only one study that mentioned serious adverse events, but the details and the numbers were not available [36]. No other serious adverse events were reported in any other study.

### Secondary outcomes

#### Achievement of BP control goals at short-term

There was insufficient evidence to determine the effects of nurse-led intervention on BP control goals compared to usual care in the short term (RR 1.17; 95%CI = 1.00,1.37; *p* = 0.05; 9 studies; 2,063 participants; *I*^2^ = 64%; τ^2^ = 0.04; Figure S2.1-b).

#### Serious adverse events at short-term

Serious adverse events were not reported in any of the included studies.

#### Average change from baseline of SBP/DBP at long and short-term

The reductions in SBP and DBP were greater in the nurse-led intervention group compared to usual care at both the long-term (MD of SBP = -2.33 mmHg; 95% CI = -3.84, -0.81; *p* = 0.003; 14 studies; 4,910 participants; Figure S2.2-a, and MD of DBP = -1.96 mmHg; 95% CI -3.10, -0.83; *p* = 0.0007; 11 studies; 2,901 participants; Figure S2.3-a) and the short-term (MD of SBP = -4.46 mmHg; 95% CI -6.32, -2.60; *p* < 0.00001; 25 studies; 4,331 participants; Figure S2.2-b, and MD of DBP = -3.31 mmHg, 95% CI = -4.54, -2.09; *p* < 0.00001; 22 studies; 2,682 participants; Figure S2.3-b).

#### Antihypertensive drug prescriptions at long and short term

One study reported long-term effects with about 90% of participants prescribed antihypertensive drugs at follow-up, compared to about 50% at baseline; however this improvement was seen both in the intervention and the control arms [38]. Two studies reported short-term effects [45, 46]. Participants prescribed antihypertensive drugs in the intervention group went from 70 to 96% [45]. However, in another study, neither group changed the percentage of antihypertensive drug prescriptions [46]. We aimed to report post-intervention antihypertensive drug prescription data. However, it remains unknown, as this information was not provided by any study.

#### Medication adherence at long and short term

Three studies reported long-term outcomes of medication adherence [36–38]. No significant differences were found in long-term effects of medication adherence (RR = 1.04; 95% CI = 0.99, 1.10; *p* = 0.12; 3 studies; 558 participants; *I*^2^ = 0%; τ^2^ = 0.00; Figure S2.4). However, medication adherence was defined by each author, such as self-report [37], pill count [38], and the medication possession into pillbox [36].

Three studies reported short-term effects of drug adherence [45, 47, 66]. The post-medication adherence rate was 80.5% in the intervention group. Meanwhile, it was 69.2% in the usual care group [45]. From available studies, 95.6% of all participants adhered to their doses [47]. Medication adherence was reported with Medication adherence self-efficacy scale short form (MASES-SF) [66]. We could not analyze these because of different ways of reporting at short-term.

#### Incidence of hypertensive complications including cardiovascular events and strokes at long and short term

No study reported the incident rate of hypertensive complications, including cardiovascular events or strokes in the included studies. Some participants were diagnosed and dropout as diabetic or hypothyroidism [66].

#### Total mortality at long and short term

**Fig. 3:**
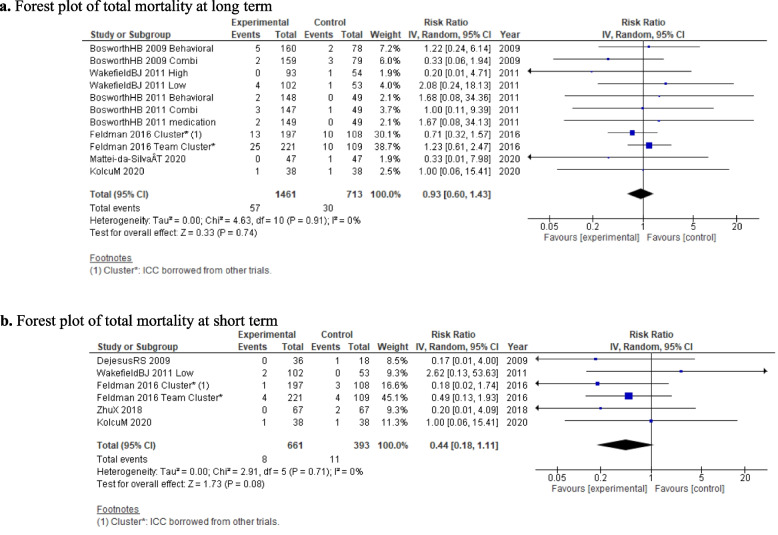
**a **Forest plot of total mortality at long term. **b** Forest plot of total mortality at short term

There was insufficient evidence to demonstrate the impact of the nurse-led intervention on mortality compared to usual care for both the long term (RR = 0.93; 95% CI = 0.60, 1.43; *p* = 0.74; 6 studies; 2,174 participants; *I*^2^ = 0%; *τ*^2^ = 0.00; Fig. 3a) and the short term (RR = 0.44; 95% CI = 0.18, 1.11; *p* = 0.08; 5 studies; 1,054 participants; *I*^2^ = 0%; *τ*^2^ = 0.00; Fig. 3b).

### Sensitivity analyses

There was insignificant evidence to determine the effects of the achievement of BP control goals at the long term when including only low risk of bias studies (RR = 1.03; 95% CI = 0.89, 1.19; *p* = 0.71; 1 study; 591 participants; *I*^2^ = 0%; *τ*^2^ = 0.00; Figure S4.1). The results of the sensitivity analyses including dropouts were consistent with the primary analysis (RR = 1.08; 95% CI = 1.02, 1.14; *p* = 0.010; 2,833 participants; *I*^2^ = 0%; *τ*^2^ = 0.00; Figure S4.2). The results of sensitivity analyses excluding borrowed ICCs also were consistent with the primary analysis (Figure S4.3). The results of sensitivity analyses excluding borrowed ICCs for the average change from baseline of SBP and DBP at the long-term were consistent with the primary analysis (Figure S4.4 and Figure S4.5). Results excluding borrowed ICCs for total mortality at long and short term were consistent with the primary analyses (Figure S4.6-a and Figure S4.6-b).

### Risk of bias in studies

**Fig. 4:**
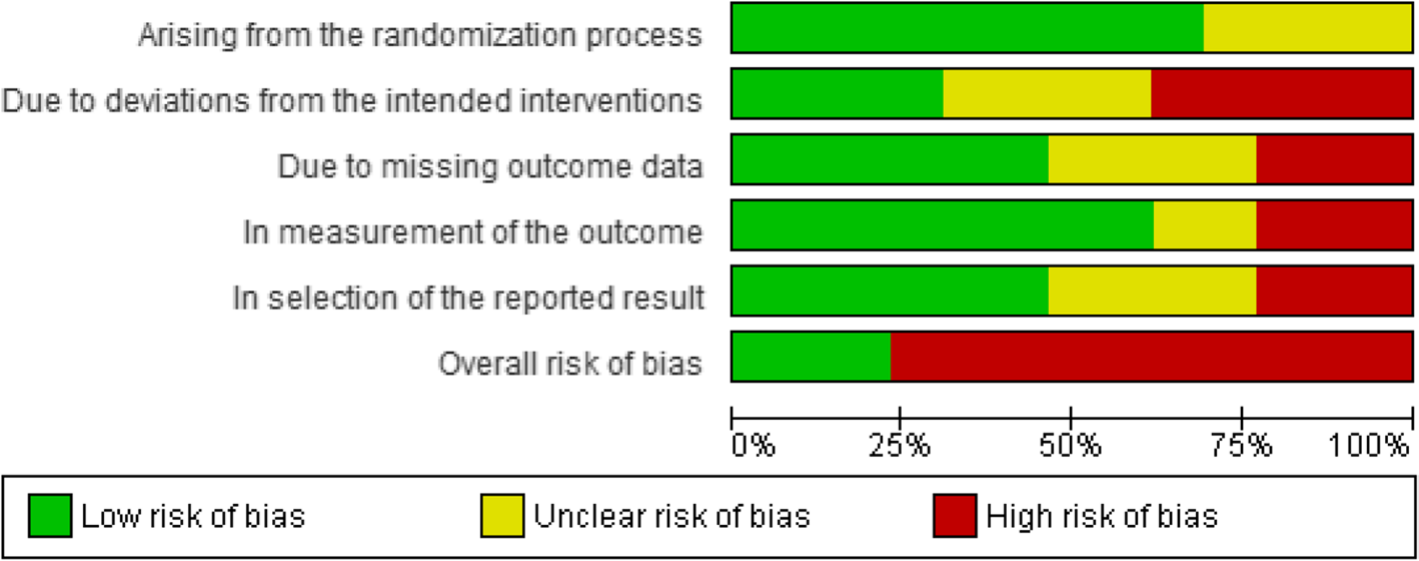
Risk of bias graph: review authors' judgements about each risk of bias item presented as percentages across all included studies about the primary outcome

Figure 4 shows the risk of bias of the included studies for the primary outcome, achievement of BP control goals at the long term. The proportion of studies rated at low risk of bias for each domain was as follows: 69.2% for risk of bias arising from the randomization process, 30.8% for bias due to deviations from the intended interventions, 46.2% for risk of bias due to missing outcome data, 61.5% for risk of bias in the measurement of the outcome, and 46.2% for risk of bias in the selection of the reported result. Finally, we assessed 23.1% of the studies at low overall risk of bias. Only one study was at overall low risk of bias [25]. See Figure S1 in the Supplemental Figures for the details of the risk of bias summary at each intervention in the primary outcome of the study.

### Reporting biases

The funnel plots are shown in Figures S5.1-a to S5.4-b and were visually symmetrical. The result of Egger’s test for continuous outcomes did not indicate reporting biases (See Table S3.)

## Discussion

Comparing nurse-led intervention plus usual physician care against usual care only, the present study found that (1) the proportion of patients achieving BP control goals was greater for nurse-led intervention at long-term, (2) the average reduction from baseline of SBP and DBP were greater for nurse-led intervention at both the short- and long-term, (3) there was insufficient evidence to determine a difference in mortality between the two methods of care, and (4) there were almost no trials reporting on serious adverse events.

The present study adds to the extant literature by identifying the benefits of nurse-led interventions to achieve BP control goals at long-term. The primary outcome of this study at long-term follow-up demonstrated lower heterogeneity in comparison with previous studies [7]. This may be due to our inclusion criteria clearly comparing nurse-led care plus usual care by physicians with usual care only. However, no benefit was found in terms of BP control goals at short term. Nurse interventions also include changing health risk behaviors for people with hypertension using behavioral management programs. Nurses need to assess patient stages of change [67], and work with them accordingly. Therefore, nursing care can take more time to achieve effectiveness, as many patients are ambivalent about changing their behaviors. Thus, we suggest that importance should be placed on longer-term targets for achieving BP control goals. In addition, the lack of evidence for the impact of nurse-led interventions on total mortality at both the short- and long-term follow-ups support similar existing systematic reviews [6].

The present study provides several contributions to the literature base. First, a prior review suggested that BP should be lowered slowly to reach the target BP over a few months [5], but did not specify how long this should take. We determined the evidence distinguishing between the short and long term. It is reasonable to expect meaningful intervention effects of nurse-led interventions to begin around seven months, as lifestyle modification and improving adherence takes time. Second, not only did we investigate efficacy but also the safety associated with the primary outcome. Previous reviews did not investigate adverse events [7, 11]. We explored serious adverse events, including death. However, we did not find sufficient evidence because adverse events were poorly reported in the included RCTs. Therefore, we suggest better safety outcome reporting for nurse-led intervention studies. We may need a guideline to collect and report safety outcomes in such RCTs. Finally, we expanded our search beyond English, while previous reviews were limited to only English. Thus, we were able to include two additional articles in Japanese.

Our study has some limitations. First, the statistical power was low because we divided the durations into short- and long-term outcomes. Thus, the number of studies in each analysis was smaller in comparison with the previous meta-analysis which did not distinguish between the durations [11]. However, the number of included studies was more than in the previous review [11]. We also found similar results for the effects of nurse-led intervention as the previous meta-analysis [11]. We highlight that the purpose of our study was originally to conduct meta-analyses by duration. We believe that distinguishing between durations is clinically meaningful. Second, there were some studies we were unsure if we should include because the primary care settings were different among countries. For example, some people see secondary or tertiary hospitals as the first step in Japan. We contacted the original authors in various countries but had to make our own decision among multiple reviewers when inquiries were not addressed. We could not divide the settings clearly for some studies. These may not have been proper primary care settings, but were included. Third, the types of nurse-led intervention are diverse, and it is difficult to distinguish from which intervention draws which effect. There were many kinds of care, and we included all nurse-led interventions. We could not distinguish between these care delivery methods, as they may include multiple elements, complex combination, and comprehensive approaches, such as medication management, diets, and weight control. Yet, the heterogeneity between the studies was low. Fourth, there are many factors that affect achievement of BP goal levels that include not only the BP target, but also the starting BP level and duration of hypertension. Our eligibility was based to the BP targets by the original authors. Therefore, in some cases there were no baseline BP levels reported and/or no reports of hypertension duration. Thus, nurse researchers may require paying attention to interpret our findings and need further investigations. Fifth, different measurements were used between studies. Those different criteria or measurements may have major impact on the results of BP control, the reduction in SBP/DBP, medication adherence and drug prescriptions. More evidence is required and we recommend future studies to report on these outcomes. Sixth, our results may not generalize to all countries and healthcare systems. Most studies included were from the USA, but nursing care varies between countries. For example, nurses in some countries cannot prescribe medications. Thus, external validity should be interpreted within the practices of individual countries. Finally, we used DeepL Translator to translate other languages into English at the phase of screening. DeepL appears reasonably accurate for technical papers, as, for example, it has been reported that the machine translation for a medical article from Japanese to English using DeepL Translator had “the mean ± SDs of the match rate for the entire article: 94.0 ± 2.9%” [68]. To assure accuracy, however, we eventually asked the native language users to check the articles.

## Conclusion

Usual physician care plus nurse-led intervention may be more effective for the achievement of long-term BP control goals compared to usual care alone. The current research base in nurse-led interventions for BP control lacks important reporting of adverse events, limiting the clinical interpretation. Additionally, more studies examining different BP targets are needed to improve the current understanding of the effects. To achieve BP control, nurse-led care is an important complement to usual physician care in primary care settings.

### Supplementary Information

**Supplementary Material 1.**

## Data Availability

The datasets used and/or analysed during the current study are available from the corresponding author on reasonable request.

## Abbreviations

BP: Blood pressure
CI: Confidence intervals
DBP: Diastolic blood pressure
ICC: Intra-cluster (or intraclass) correlation coefficient
MD: Mean difference
RCTs: Randomized controlled trials
RoB 2.0: Risk of Bias Tool Ver. 2.0
RR: Risk ratios
SBP: Systolic blood pressure
SDs: Standard deviations
