# Supplementary material for: The short and long-term efficacy of nurse-led interventions for improving blood pressure control in people with hypertension in primary care settings: a systematic review and meta-analysis
Source: BMC Prim Care. 2024 Apr 27;25:143. doi: 10.1186/s12875-024-02380-x (PMC11056068; doi:10.1186/s12875-024-02380-x)
Supplement: Supplementary file 1 — Supplementary Material 1. [file 12875_2024_2380_MOESM1_ESM.docx]

**Supplemental Figures and Figure Legends**

Risk of bias in studies

Figure S1. Risk of bias summary: review authors' judgements about each risk of bias item at each intervention for primary outcome of study.

Meta-analysis

*Primary outcome*

Figure S2.1-a Forest plot of the achievement proportion of the goals at the long-term

*Secondary outcomes*

Figure S2.1-b Forest plot of the achievement proportion of the goals at the short-term

Figure S2.2-a Forest plot of SBP at the long-term

Figure S2.2-b Forest plot of SBP at the short-term

Figure S2.3-a Forest plot of DBP at the long-term

Figure S2.3-b Forest plot of DBP at the short-term

Figure S2.4 Forest plot of medication adherence at the long-term

Subgroup analysis

*Primary outcome*

Figure S3.1 The achievement proportion of the goals at the long-term (the district of recruitment)

Figure S3.2 The achievement proportion of the goals at the long-term (the settings of care)

Sensitivity analysis

*Primary outcome*

Figure S4.1 The achievement proportion of the goals at the long-term (Low risk of biases)

Figure S4.2 The achievement proportion of the goals at the long-term (included dropout)

Figure S4.3 The achievement proportion of the goals at the long-term (excluded the studies borrowed ICC from others)

*Secondary outcomes*

Figure S4.4 SBP at the long-term (excluded the studies borrowed ICC from others)

Figure S4.5 DBP at the long-term (excluded the studies borrowed ICC from others)

Figure S4.6-a Mortality at the long-term (excluded the studies borrowed ICC from others)

Figure S4.6-b Mortality at the short-term (excluded the studies borrowed ICC from others)

Reporting biases

*Primary outcome*

Figure S5.1-a Funnel plot of the achievement proportion of the goals at the long-term

*Secondary outcomes*

Figure S5.1-b Funnel plot of the achievement proportion of the goals at the short-term

Figure S5.2-a Funnel plots of SBP at the long-term

Figure S5.2-b Funnel plot of SBP at the short-term

Figure S5.3-a Funnel plot of DBP at the long-term

Figure S5.3-b Funnel plot of DBP at the short-term

Figure S5.4-a Funnel plot of mortality at the long-term

Figure S5.4-b Funnel plot of mortality at the short-term

**Supplemental Tables and supporting information**

Table S1. Search sources and search strategies; the English language only

Table S2. Overview of our outcomes in the included studies

Table S3. The results of Egger’s test

Risk of bias in studies

**
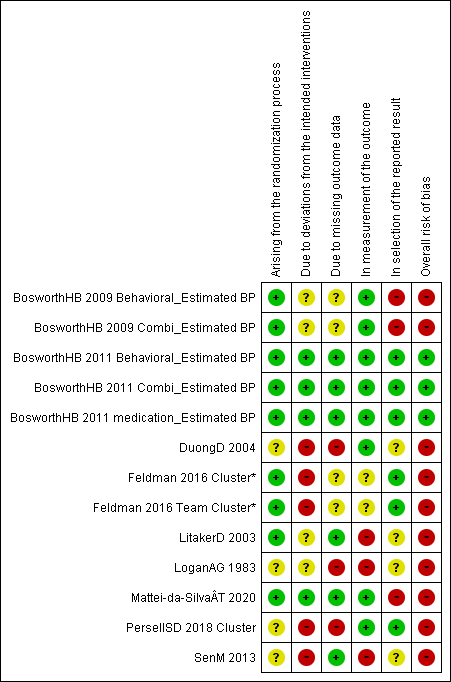
**

Figure S1.

Risk of bias summary: review authors' judgements about each risk of bias item at each intervention for primary outcome of study.

^*^ We showed “Cluster*” in the studies borrowed ICC from other trials. It is not relevant for the evaluation of Risk of bias.

Meta-analysis

*Primary outcome*

*
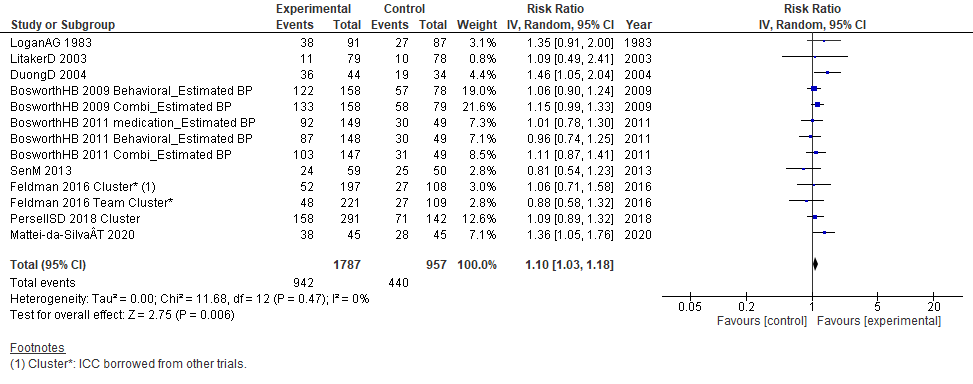
*

Figure S2.1-a The achievement proportion of the goals at the long-term

*Secondary outcomes*


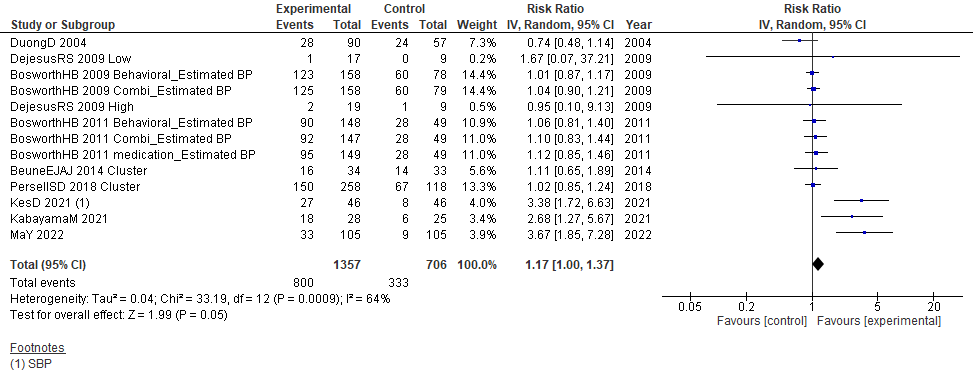


Figure S2.1-b Forest plot of the achievement proportion of the goals at the short-term


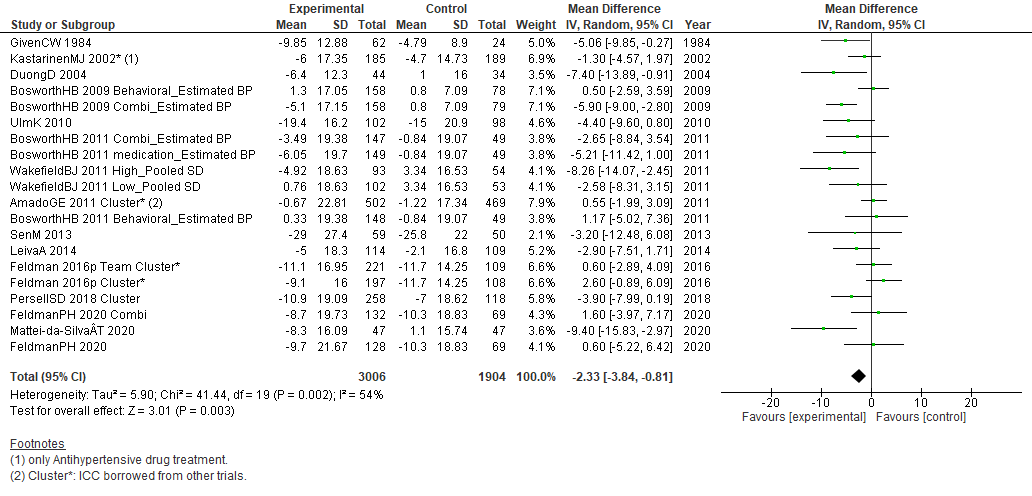


Figure S2.2-a Forest plot of SBP at the long-term


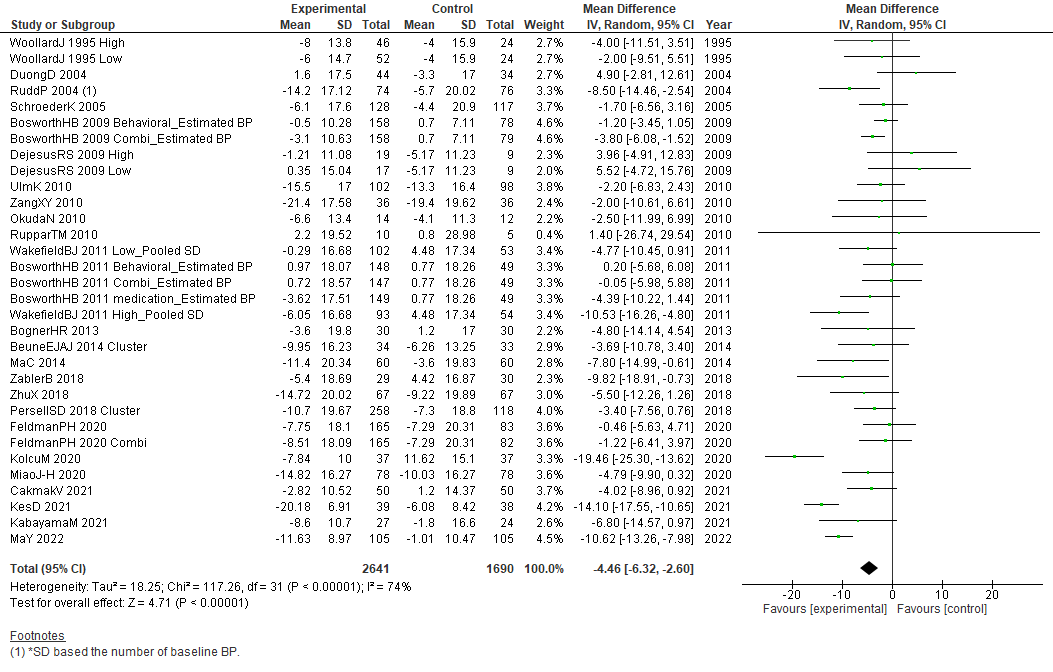


Figure S2.2-b Forest plot of SBP at the short-term


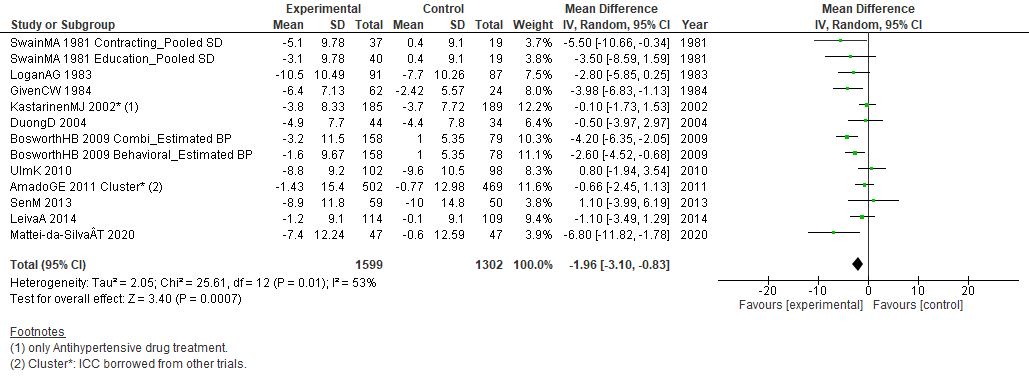


Figure S2.3-a Forest plot of DBP at the long-term


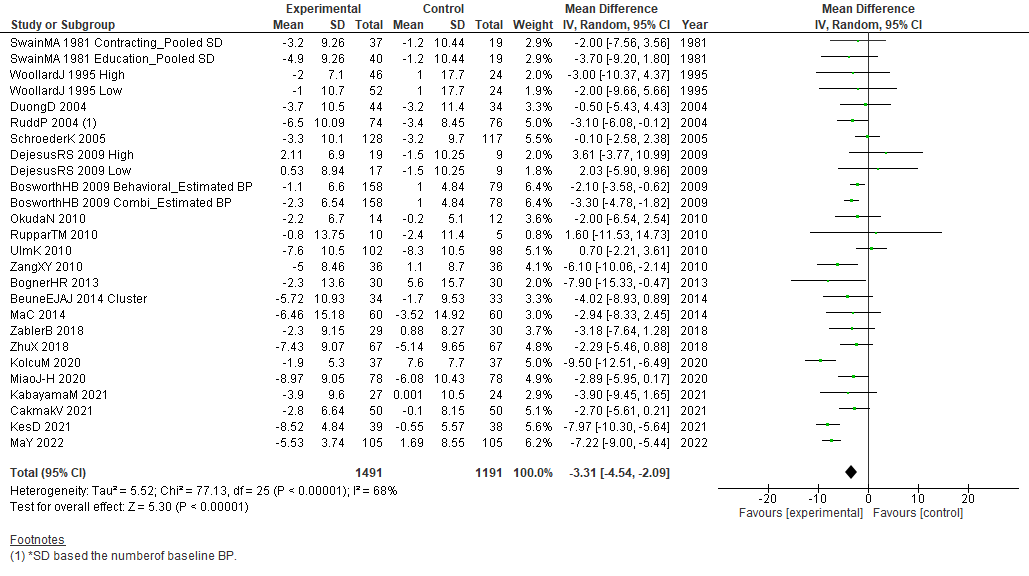


Figure S2.3-b Forest plot of DBP at the short-term


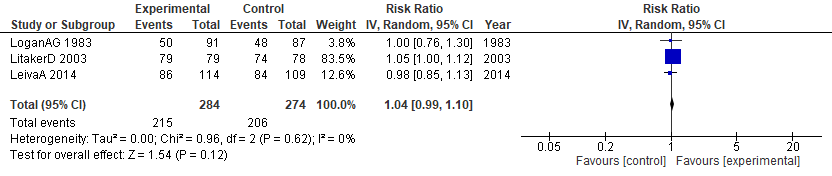


Figure S2.4 Forest plot of medication adherence at the long-term

*Subgroup analysis*


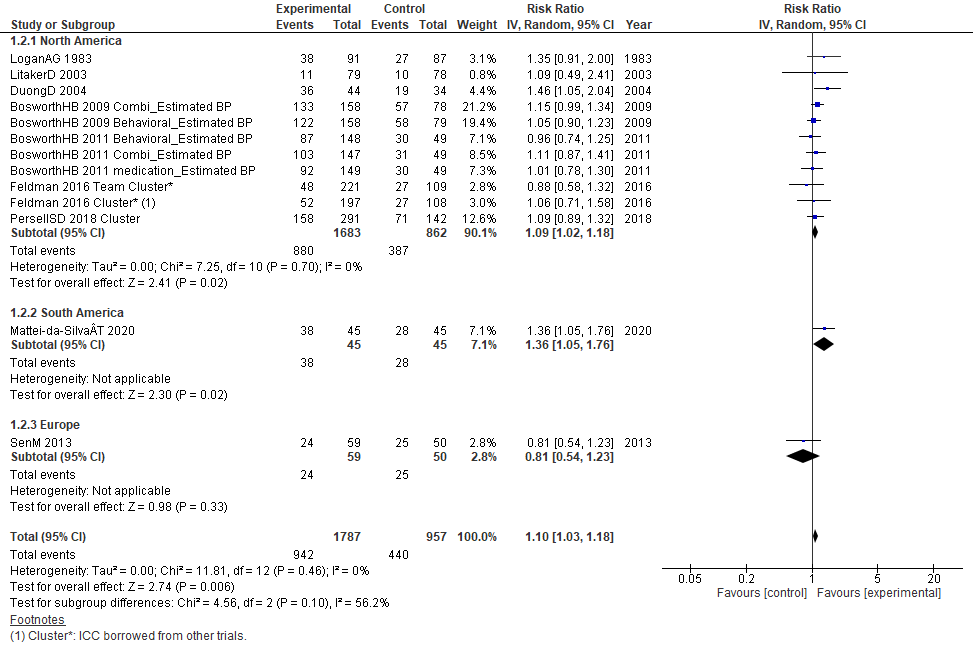


Figure S3.1 The achievement proportion of the goals at the long-term (the region of recruitment)


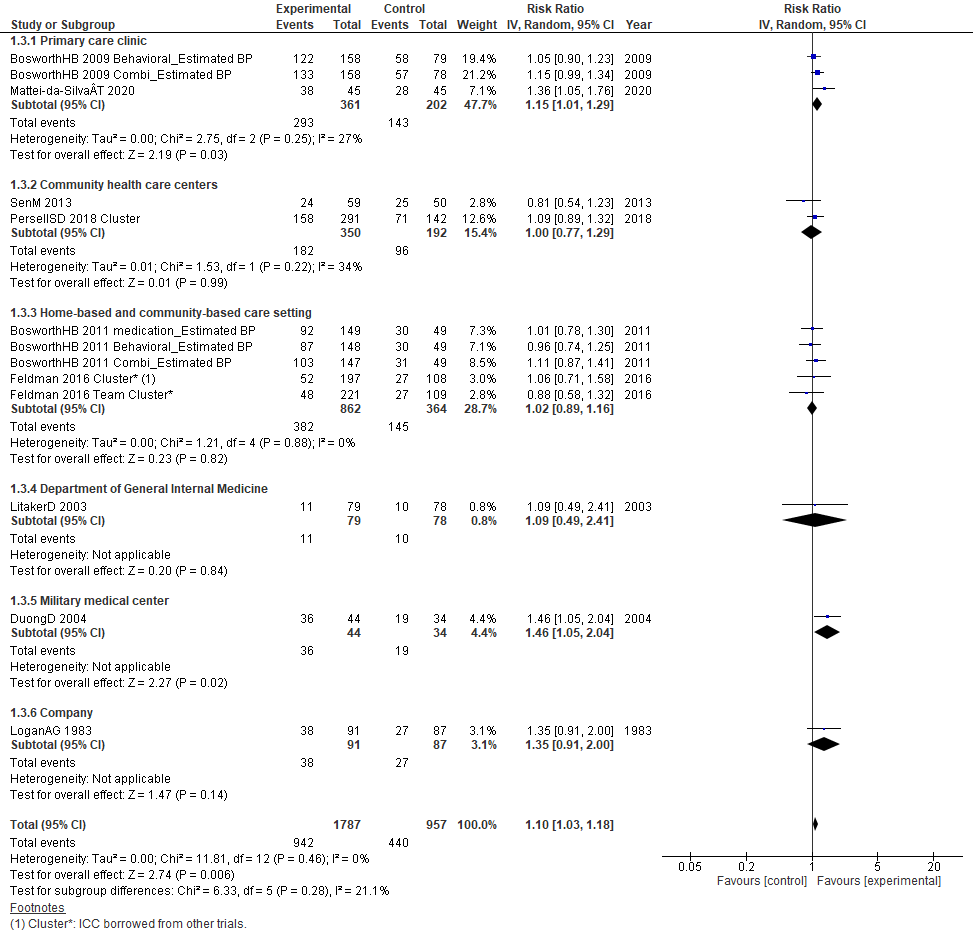


Figure S3.2 The achievement proportion of the goals at the long-term (settings)

*Sensitivity analysis*


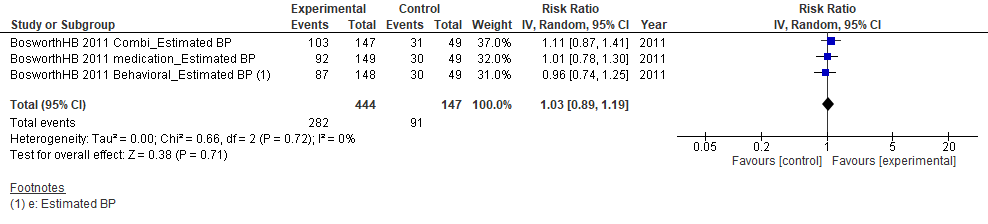


Figure S4.1 The achievement proportion of the goals at the long-term (Low risk of biases)


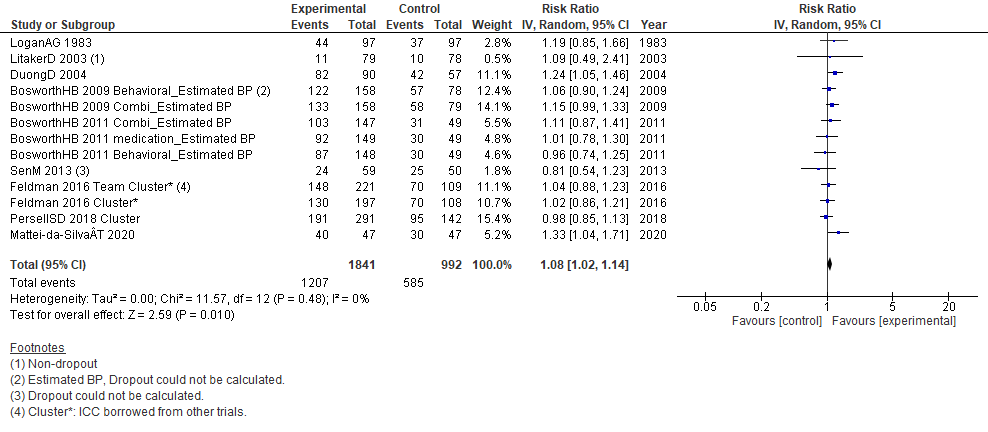


Figure S4.2 The achievement proportion of the goals at the long-term (included dropout)


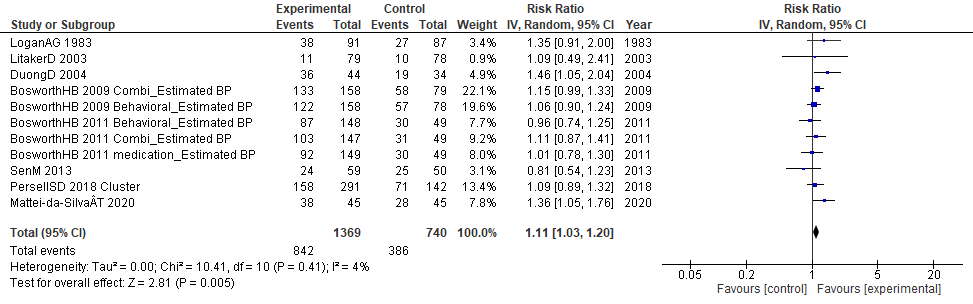


Figure S4.3 The achievement proportion of the goals at the long-term (excluded the studies borrowed ICC from others)

*Secondary outcomes*


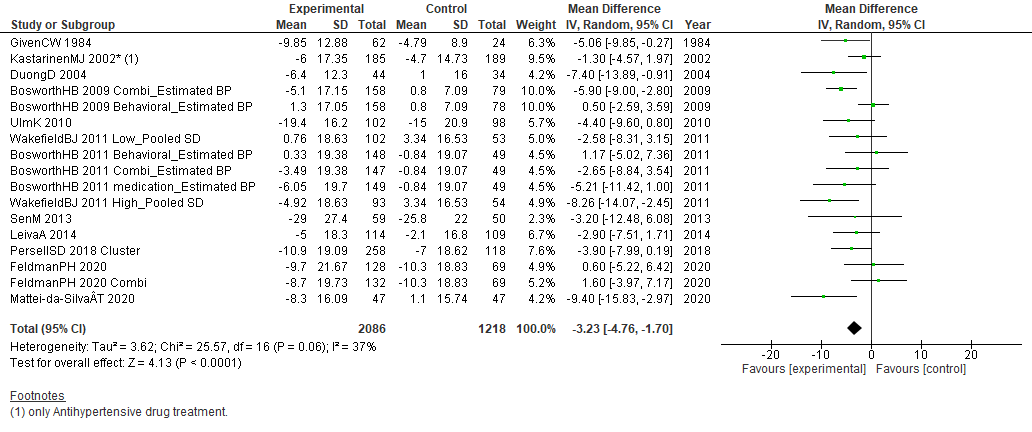


Figure S4.4 SBP at the long-term (excluded the studies borrowed ICC from others)


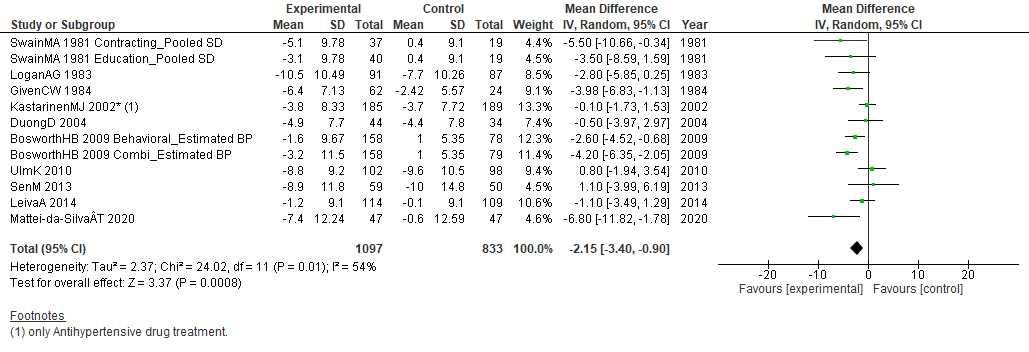


Figure S4.5 DBP at the long-term (excluded the studies borrowed ICC from others)


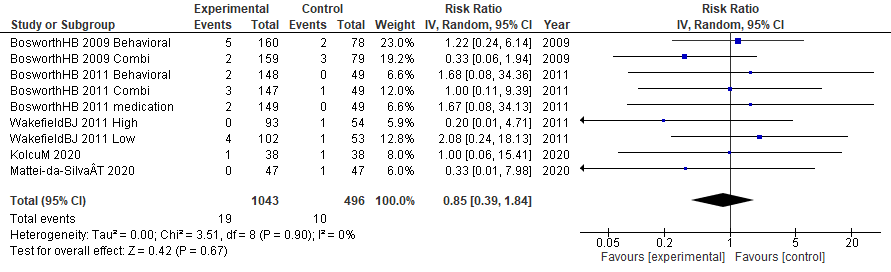


Figure S4.6-a Mortality at the long-term (excluded the studies borrowed ICC from others)


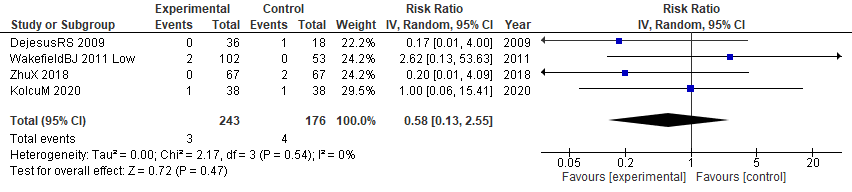


Figure S4.6-b Mortality at the short-term (excluded the studies borrowed ICC from others)

Reporting biases

*Primary outcome*


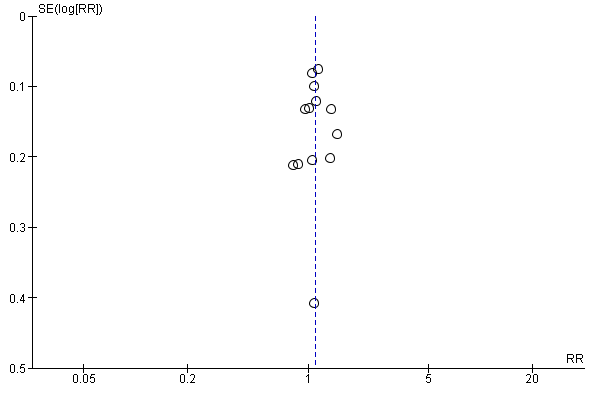


Figure S5.1-a Funnel plot of the achievement proportion of the goals at the long-term

*Secondary outcomes*


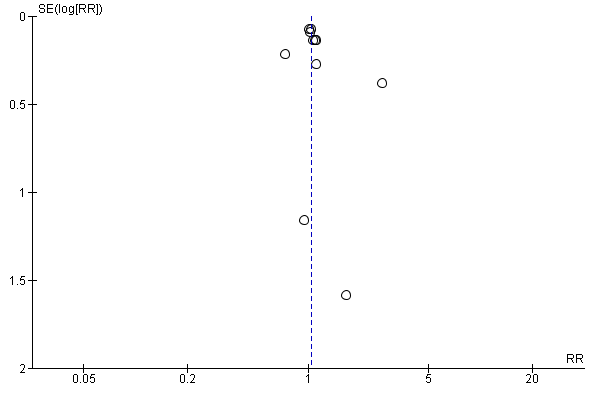


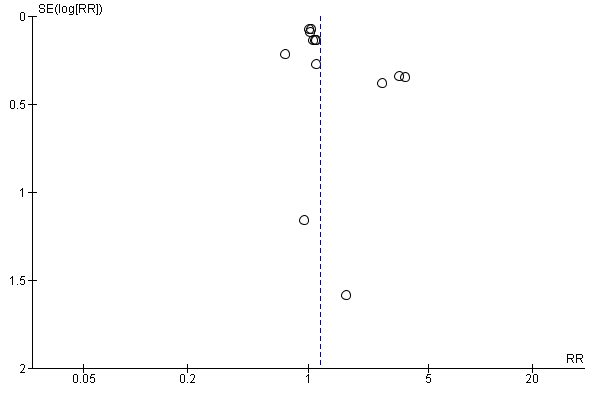


Figure S5.1-b Funnel plot of the achievement proportion of the goals at the short-term


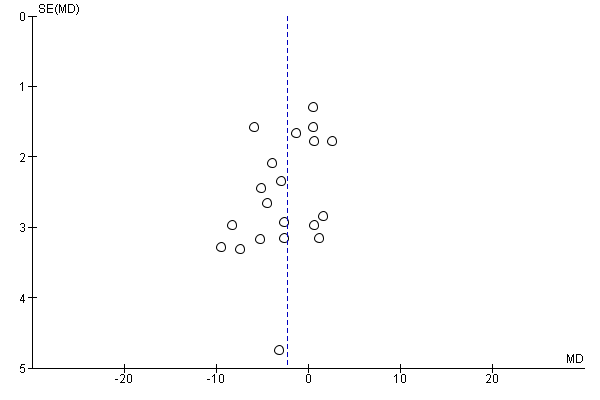


Figure S5.2-a Funnel plots of SBP at the long-term


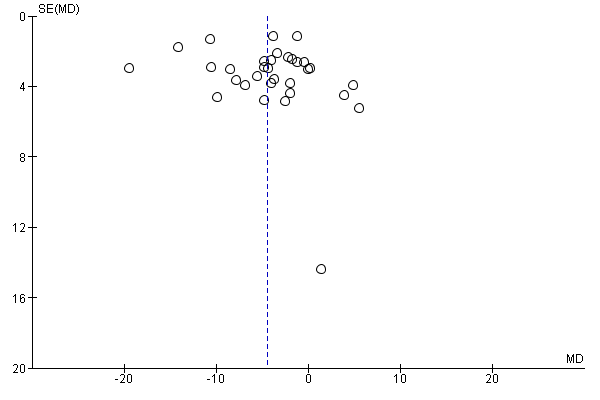


Figure S5.2-b Funnel plot of SBP at the short-term


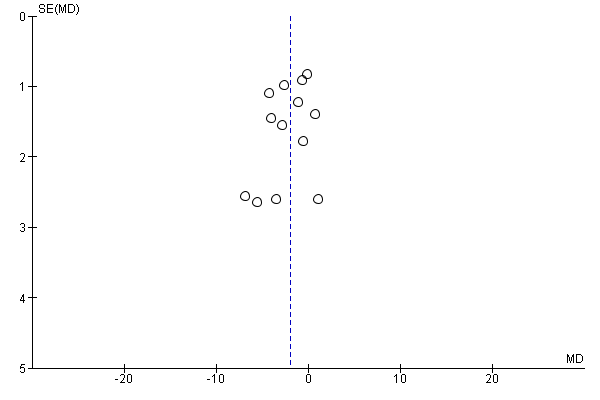


Figure S5.3-a Funnel plot of DBP at the long-term


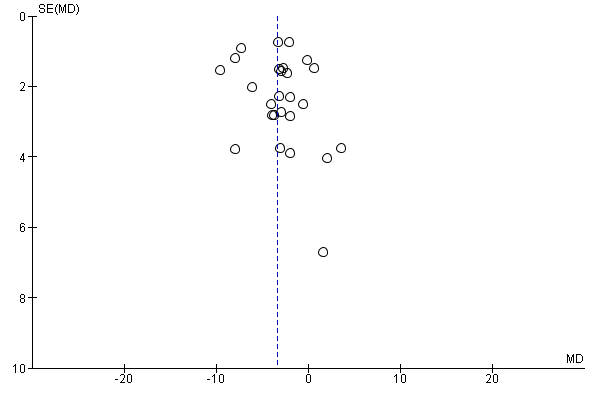


Figure S5.3-b Funnel plot of DBP at the short-term


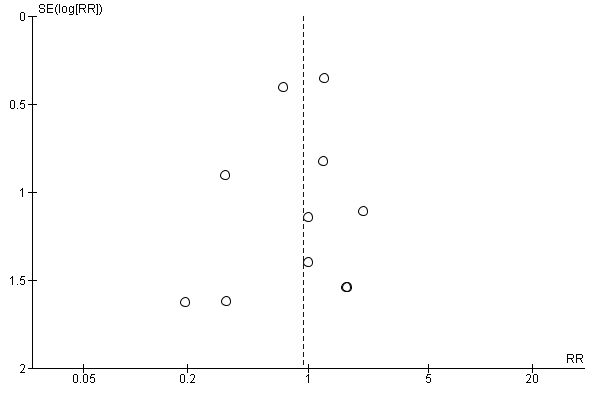


Figure S5.4-a Funnel plot of mortality at the long-term


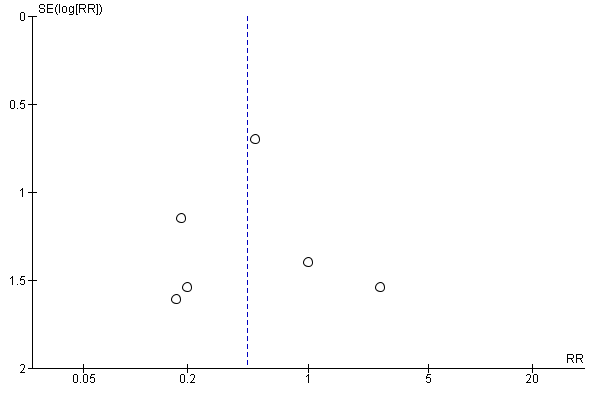


Figure S5.4-b Funnel plot of mortality at the short-term

**Supplemental Tables and supporting information**

Table S1. Search sources and search strategies

| 1. the Cochrane Central Register of Controlled Trials (CENTRAL) on The Cochrane Library |
| --- |
| #1 MeSH descriptor: [Hypertension] explode all trees  #2 MeSH descriptor: [Antihypertensive Agents] explode all trees  #3 MeSH descriptor: [Blood Pressure] explode all trees  #4 (blood NEXT pressure):ti  #5 hypertens*  #6 {OR #1-#5}  #7 MeSH descriptor: [Physicians] explode all trees  #8 MeSH descriptor: [Patient Care Management] explode all trees  #9 MeSH descriptor: [Patient Care Planning] explode all trees  #10 MeSH descriptor: [Patient Care Team] explode all trees  #11 MeSH descriptor: [Patient Education as Topic] explode all trees  #12 MeSH descriptor: [Patient Participation] explode all trees  #13 MeSH descriptor: [Ambulatory Care Information Systems] explode all trees  #14 MeSH descriptor: [Feedback] explode all trees  #15 MeSH descriptor: [Information Systems] explode all trees  #16 MeSH descriptor: [Management Information Systems] explode all trees  #17 MeSH descriptor: [Decision Support Systems, Clinical] explode all trees  #18 MeSH descriptor: [Decision Making, Computer-Assisted] explode all trees  #19 MeSH descriptor: [Reminder Systems] explode all trees  #20 MeSH descriptor: [Practice Guidelines as Topic] explode all trees  #21 MeSH descriptor: [Guidelines as Topic] explode all trees  #22 MeSH descriptor: [Medical Records] explode all trees  #23 MeSH descriptor: [Outcome and Process Assessment, Health Care] explode all trees  #24 MeSH descriptor: [Medical Records Systems, Computerized] explode all trees  #25 MeSH descriptor: [Primary Health Care] explode all trees  #26 MeSH descriptor: [Physicians, Family] explode all trees  #27 MeSH descriptor: [Health Behavior] explode all trees  #28 remind*  #29 motiv*  #30 MeSH descriptor: [Patient Care] explode all trees  #31 MeSH descriptor: [Guideline Adherence] explode all trees  #32 MeSH descriptor: [Behavior Therapy] explode all trees  #33 MeSH descriptor: [Counseling] explode all trees  #34 counsel*  #35 MeSH descriptor: [Motivation] explode all trees  #36 self NEXT monitor*  #37 ((patient* or program*) NEAR/6 (educat* or manage* or train* or teach*))  #38 self NEXT manag*  #39 ((manag* or monitor*) NEAR/6 (hypertension or blood pressure))  #40 MeSH descriptor: [Health Promotion] explode all trees  #41 MeSH descriptor: [Health Education] explode all trees  #42 reward* or incentiv*  #43 uncontrol*  #44 MeSH descriptor: [Self Care] explode all trees  #45{OR #7-#44}  #46 MeSH descriptor: [Practice Patterns, Nurses'] explode all trees  #47 MeSH descriptor: [Primary Nursing] explode all trees  #48 MeSH descriptor: [Nursing Care] explode all trees  #49 MeSH descriptor: [Nurse Practitioners] explode all trees  #50 MeSH descriptor: [Nurse Clinicians] explode all trees  #51 MeSH descriptor: [Nursing] in all MeSH products  #52 nurse  #53{OR #46-#52}  #54 #6 AND #45 AND #53  #55 #54in Trials |
| 1. PubMed |
| ((((Hypertension[MeSH Terms]) OR (Antihypertensive Agents[MeSH Terms])) OR (Blood Pressure[MeSH Terms])) AND (((((((((physicians[MeSH Terms]) OR (Patient Care Management[MeSH Terms])) OR (Patient Care Planning[MeSH Terms])) OR (patient care team[MeSH Terms])) OR (Patient Education as Topic[MeSH Terms])) OR (Patient Participation[MeSH Terms])) OR ((((((((((Ambulatory Care Information Systems[MeSH Terms]) OR (Feedback[MeSH Terms])) OR (Information Systems[MeSH Terms])) OR (Management Information Systems[MeSH Terms])) OR (decision support systems, clinical[MeSH Terms])) OR (decision making, computer assisted[MeSH Terms])) OR (Reminder Systems[MeSH Terms])) OR (Practice Guidelines as Topic[MeSH Terms])) OR (Guidelines as Topic[MeSH Terms])) OR (Medical Records[MeSH Terms]))) OR ((((((((((outcome and process assessment health care[MeSH Terms]) OR (medical records systems, computerized[MeSH Terms])) OR (Primary Health Care[MeSH Terms])) OR (physicians, family[MeSH Terms])) OR (health behavior[MeSH Terms])) OR (remind*)) OR (motiv*)) OR (patient care[MeSH Terms])) OR (guideline adherence[MeSH Terms])) OR (behavior therapy[MeSH Terms]))) OR ((((((((counseling[MeSH Terms]) OR (counsel*)) OR (Motivation[MeSH Terms])) OR (Health Promotion[MeSH Terms])) OR (Health Education[MeSH Terms])) OR (reward* or incentiv*)) OR (uncontrol*)) OR (self care[MeSH Terms])))) AND (((((((practice patterns, nurse's[MeSH Terms]) OR (primary nursing[MeSH Terms])) OR (nursing care[MeSH Terms])) OR (nurse practitioners[MeSH Terms])) OR (nurse clinicians[MeSH Terms])) OR (nursing care)) OR (nurse)) |
| 1. CINAHL |
| S1 MH hypertension OR MH antihypertensive agents OR MH blood pressure OR hypertens*  S2 MH ( physicians or doctors ) OR MH patient care management OR MH patient care planning OR MH patient care team OR MH patient education as topic OR MH patient participation OR MH information systems OR MH feedback OR MH management information systems OR MH decision support systems, clinical OR MH ( decision making or decision-making or decision making process or decision-making process ) OR MH ( reminder systems or clinical reminders )  S3 MH practice guidelines as topic OR MH guidelines OR MH medical records OR MH ( outcome and process assessment, health care ) OR MH medical records systems OR MH primary health care OR MH physicians, family OR MH health behavior OR MH patient care OR MH guideline adherence OR MH behavior therapy OR MH counseling  S4 remind* OR motiv* OR counsel* OR self N monitor* OR ( ((patient* or program*) N6 (educat* or manage* or train* or teach*)) ) OR self N manag* OR ( ((manag* or monitor*) N6 (hypertension or blood pressure)) )  S5 MH motivation OR MH health promotion OR MH health education OR ( reward* or incentiv* ) OR uncontrol* OR MH self care  S6 S2 OR S3 OR S4 OR S5  S7 MH practice pattern, nurses' OR MH primary nursing OR MH nursing care OR MH nurse clinicians OR nurse practitioners OR nursing care OR nurse  S8 S1 AND S6 AND S7  S9 S1 AND S6 AND S7 |

Table S2. Overview of our outcomes in the included studies

| Study | Primary outcomes | | Secondary outcomes | | | | | | | | | | | | |
| --- | --- | --- | --- | --- | --- | --- | --- | --- | --- | --- | --- | --- | --- | --- | --- |
| Author, Year of publication | (1) Achievement of BP control goals at the long-term | (2) Serious adverse events at the long-term | (1) Achievement of BP control goals at the short-term | (2) Serious adverse events at the short-term | (3)  Change of SBP from baseline | | Change of DBP from baseline | | (4)  Prescribing antihypertensive drugs | | (5)  Drug adherence | | (6) Incidence of hypertensive complications including cardiovascular events and strokes | (7)  Total mortality | |
|  |  |  |  |  | long-term | short-term | long-term | short-term | long-term | short-term | long-term | short-term | Both the short and long terms | long-term | short-term |
| AmadoGE, 2011 | - | - | - | - | + | - | + | - | - | - | - | - | - | - | - |
| BeuneEJAJ, 2014 | - | - | + | - | - | + | - | + | - | - | - | - | - | - | - |
| BognerHR, 2013 | - | - | - | - | - | + | + | + | - | - | - | - | - | - | - |
| BosworthHB, 2009 | + | - | + | - | + | + | - | + | - | - | - | - | - | + | - |
| BosworthHB, 2011  CakamakV, 2021 | +  - | -  - | +  - | -  - | +  - | +  + | -  - | -  + | -  - | -  - | -  - | -  - | -  - | +  - | -  - |
| DejesusRS, 2009 | - | - | + | - | - | + | - | + | - | - | - | - | - | - | + |
| DuongD, 2004 | + | - | + | - | + | + | + | + | - | - | - | - | - | - | - |
| FeldmanPH, 2020 | - | - | - | - | + | + | - | - | - | - | - | - | - | - | + |
| FeldmanPH, 2016 | + | - | - | - | + | - | - | - | - | - | - | - | - | + | - |
| GivenCW, 1984 | - | - | - | - | + | - | + | - | - | - | - | - | - | - | - |
| KabayamaM, 2021 | - | - | + | - | - | + | - | + | - | - | - | - | - | - | - |
| KastarinenMJ, 2002 | - | - | - | - | + | - | + | - | - | - | - | - | - | - | - |
| KolcuM, 2020  KesD, 2021 | -  - | -  - | -  + | -  - | -  - | +  + | -  - | +  + | -  - | -  - | -  - | -  + | -  + | +  - | +  - |
| LeivaA, 2014 | - | + | - | - | + | - | + | - | - | - | + | - | - | - | - |
| LitakerD, 2003 | + | - | - | - | - | - | - | - | - | - | + | - | - | - | - |
| LoganAG, 1983 | + | - | - | - | - | - | + | - | + | - | + | - | - | - | - |
| MaC, 2014  MaY, 2022 | -  - | -  - | -  + | -  - | -  - | +  + | -  - | +  + | -  - | -  - | -  - | -  - | -  - | -  - | -  - |
| MatteidaSilvaÂT, 2020 | + | - | - | - | + | - | + | - | - | - | - | - | - | + | - |
| MiaoJ-H, 2020 | - | - | - | - | - | + | - | + | - | - | - | - | - | - | - |
| OkudaN, 2010 | - | - | - | - | - | + | - | + | - | - | - | - | - | - | - |
| PersellSD, 2018 | + | - | + | - | + | + | - | - | - | - | - | - | - | - | - |
| RuddP, 2004 | - | - | - | - | - | + | - | + | - | + | - | + | - | - | - |
| RupparTM, 2010 | - | - | - | - | - | + | - | + | - | + | - | - | - | - | - |
| SchroederK, 2005 | - | - | - | - | - | + | - | + | - | - | - | + | - | - | - |
| SenM, 2013 | + | - | - | - | + | - | + | - | - | - | - | - | - | - | - |
| SwainMA, 1981 | - | - | - | - | - | - | + | + | - | - | - | - | - | - | - |
| UlmK, 2010 | - | - | - | - | + | + | + | + | - | - | - | - | - | - | - |
| WakefieldBJ, 2011 | - | - | - | - | + | + | - | - | - | - | - | - | - | + | + |
| WoollardJ, 1995 | - | - | - | - | - | + | - | + | - | - | - | - | - | - | - |
| ZablerB, 2018 | - | - | - | - | - | + | - | + | - | - | - | - | - | - | - |
| ZangXY, 2010 | - | - | - | - | - | + | - | + | - | - | - | - | - | - | - |
| ZhuX, 2018 | - | - | - | - | - | + | - | + | - | - | - | - | - | - | + |
|  | 9 | 1 | 9 | 0 | 14 | 25 | 11 | 22 | 1 | 2 | 3 | 3 | 0 | 6 | 5 |

-: No report.

Table S3. The results of Egger’s test

| Egger's test | Intercept | Confidence Interval | t | p |
| --- | --- | --- | --- | --- |
| SBP: long-term | -1.90 | [-3.93, 0.13] | -1.97 | 0.07 |
| SBP: short-term | 0.55 | [-1.04, 2.15] | 0.71 | 0.48 |
| DBP: long-term | -1.39 | [-3.78, 1.00] | -1.28 | 0.23 |
| DBP: short-term | 0.59 | [-0.87, 2.05] | 0.84 | 0.41 |
